# Coexistence of medullary thyroid carcinoma and recurrent non-functional pituitary adenoma: a case report

**DOI:** 10.1186/s13256-018-1745-5

**Published:** 2018-08-15

**Authors:** Mohammad Bagherzadeh, Arya Aminorroaya, Jamshid Vafaeimanesh, Mohammad Reza Mohajeri-Tehrani

**Affiliations:** 1 Clinical Research Development Center, Qom University of Medical Sciences, Qom, Iran; 2 Students’ Scientific Research Center (SSRC), Tehran University of Medical Sciences, Tehran, Iran; 3 Universal Scientific Education and Research Network (USERN), Tehran, Iran; 4 Gastroenterology and Hepatology Research Center, Qom University of Medical Sciences, Qom, Iran; 5 Endocrinology and Metabolism Research Center, Endocrinology and Metabolism Clinical Sciences Institute, Tehran University of Medical Sciences, Tehran, Iran; 6 Endocrinology and Metabolism Research Institute (EMRI), Jalal-Al-Ahmad Ave, Tehran, 1411713137 Iran

**Keywords:** Pituitary adenoma, Pituitary neoplasms, Multiple endocrine neoplasia, Thyroid cancer, Medullary, Thyroid neoplasms

## Abstract

**Background:**

Medullary thyroid carcinoma and pituitary adenoma are neuroendocrine tumors and their coexistence has not been reported in the literature, previously. Medullary thyroid carcinoma is a neoplasm of the thyroid gland arising from parafollicular c-cells producing calcitonin, and pituitary adenoma is a benign hyperplasia of the cells of the pituitary gland. Coexistence of these neoplasms can be explained by being affected by simultaneous primary neoplasms or tumor-to-tumor metastasis phenomenon.

**Case presentation:**

We present the case of a 60-year-old Persian man who presented to the clinic with a chief complaint of headache for the last 2 months. His past medical history was significant for non-functional pituitary macroadenoma and medullary thyroid carcinoma and he had received a total thyroidectomy and a transsphenoidal surgery several years ago. Diagnostic evaluations revealed that the pituitary adenoma has recurred. He was well and symptom-free after the second transsphenoidal surgery for resection of the adenoma. Noticeably, investigations were negative for any form of multiple endocrine neoplasia syndromes; however, we could not rule them out definitively.

**Conclusions:**

To the best of our knowledge, it is the first case reported in the literature of a patient who has been affected by recurrent non-functional pituitary adenoma and medullary thyroid carcinoma, concomitantly. Although this association can be accidental, it emphasizes the fact that patients with a history of a neoplasm should be monitored regularly in order to diagnose and treat possible second primary cancers in a timely manner. Of note, this consideration is of great importance in patients whose first neoplasms have better prognosis and survival rates, which provide them more time to develop second primary cancers, for example, pituitary adenoma.

## Background

Medullary thyroid carcinoma (MTC) is a neuroendocrine tumor which originates from parafollicular c-cells producing calcitonin and comprises 2.1% of thyroid neoplasms. In the hereditary form, which consists of 25% of cases, MTC is associated with an autosomal dominant mutation of *RET* proto-oncogene and can be accompanied by other endocrine tumors in multiple endocrine neoplasia (MEN) 2A and 2B syndromes [1].

Pituitary adenoma (PA) is another neuroendocrine tumor with a prevalence of 16.7% in the general population based on imaging and autopsy studies. It is categorized based on size and production of hormones to macroadenoma versus microadenoma and functional versus non-functional adenoma, respectively [2]. Recurrence of this neoplasm is not an uncommon phenomenon with the prolactinoma followed by non-functional PA as the most recurrent variants [3].

Coexistence of these neoplasms can be explained by being affected by simultaneous primary neoplasms [4] or tumor-to-tumor metastasis phenomenon [5–7]. Histopathologic examination is a helpful tool for differentiating these entities. In this communication, we are going to describe a case of recurrent non-functional PA with MTC.

## Case presentation

A 60-year-old Persian man presented to the clinic with a chief complaint of headache for the last 2 months ago. He also suffered from progressive bitemporal hemianopia. His past medical history was significant for a non-functional pituitary macroadenoma which culminated in a transsphenoidal surgery (TSS) and complete resolution of symptoms, 11 years ago; however, microscopic examination of the tumor is not available, currently. Moreover, he received a total thyroidectomy because of MTC, 3 years ago. Also, he had mild hypertension controlled by anti-hypertensive drugs, with no history of hypertension crisis, and an asymptomatic kidney stone for the last 3 years. His familial history and habitual history were not remarkable. A physical examination was not significant, except for lymphadenopathy in left cervical chain.

With the impression of PA, the patient underwent brain magnetic resonance imaging (MRI). It demonstrated an isointense and high intense heterogeneous 34 × 27 mm solid mass, in T1 and T2 sequences, respectively, which was enhanced after contrast media administration. The lesion consisted of cystic and hemorrhagic centers with involvement of sella turcica and suprasellar cistern, and suprasellar extension. Moreover, it caused a mild mass effect on adjacent optic chiasm and bilateral involvement of cavernous sinuses. All of these findings suggested pituitary macroadenoma as the most probable diagnosis. Laboratory measurements failed to detect pituitary hormones in plasma; however, they had not been coupled with stimulation tests. Furthermore, level of serum prolactin was normal even after a dilution study.

The adenoma was resected through a non-complicated TSS. Post-operation MRI showed a non-enhancing center rimmed by a solid enhancing tissue consistent with tumor remnant in the sella area. Histopathologic and immunohistochemical (IHC) findings of the lesion were in favor of gonadotroph cell adenoma with a Ki-67 index of 4%; however, staining of the specimen was negative for luteinizing hormone and follicle-stimulating hormone, which confirmed the non-functional nature of the adenoma. Our patient’s symptoms, including headache and impaired visual field, were resolved remarkably after the surgery.

Further assessments were performed in order to evaluate the possible coexistence of MEN syndromes. Metabolites of cathecholamines including metanephrine, normetanephrine, and vanillylmandelic acid in 24-hour urine collections were in normal range, excluding pheochromocytoma. Furthermore, genetic testing for mutation of exons 10, 11, 13, 14, 15, and 16 of *RET* proto-oncogene was negative. Plasma calcium and parathyroid hormone levels were normal with no signs or symptoms of pancreatic adenoma (PA).

A neck ultrasonography was done due to the detected lymphadenopathy in the physical examination, which revealed a 15 × 7 mm lymph node with a bizarre shape and hilum in the left cervical chain suggestive of a metastatic lymph node. Furthermore, a 6 mm hypoechoic nodule in the left thyroid bed was detected that might be attributed to the thyroid gland remnant or local recurrence of MTC. Therefore, our patient underwent cervical lymph node dissection and surgical removal of the nodule. Consequently, histopathologic examinations delineated the nature of the lymph node and nodule as MTC metastatic lymph node and recurrence of MTC, respectively.

## Discussion and conclusions

Detecting a mass in the pituitary gland of a patient who had a past medical history of PA and MTC raises both the possibility of recurrence of PA or metastasis of MTC. In our case, histopathologic and IHC findings of the specimen confirmed the former possibility; however, there are multiple reports about metastasis of MTC to the pituitary gland, which is an unusual site of metastasis [5–7]. Therefore, we confronted the coexistence of two primary neoplasms in this patient.

There are several reports regarding patients who had simultaneous PA and MTC. Non-functional pituitary macroadenoma in conjunction with a full-blown MEN2A (MTC, parathyroid adenoma, and pheochromocytoma) has been reported in a patient with a missense mutation in exon 10 of *RET* gene [8]. In another study, Saito *et al.* reported coexistence of MTC and acromegaly in a case with a mutation of *RET* gene. The patient underwent resection of the PA that was positive for growth hormone and prolactin in an IHC study. They suggested a possible association between *RET* gene and glial cell line-derived neurotrophic factor (GDNF), based on previous animal and human studies, which may explain the association between MTC and somatotroph cell PA [4]. Notably, these cases had concomitant genetic mutations, while genetic disturbances are not likely in our patient due to results of genetic testing and negative familial history. It should be noted that we could not rule out genetic disturbances and also MEN syndromes due to considering just 6 exons of the *RET* gene, not all of them, and different penetrance of the components of MEN in a life time, respectively.

Recurrence of PA is a challenging issue due to lack of certain prognostic factors for its prediction. In the most recent meta-analysis, Roelfsema *et al.* failed to introduce risk factors of recurrence of non-functional PA [3]; however, there is evidence demonstrating the promising role of the Ki-67 index [9, 10]. Studies show that the higher the Ki-67 index of the PA, the higher the probability of recurrence, with reported cutoff values of 1.3% [9] and 2.2% [10]. Therefore, recurrence of PA in our patient is due to high Ki-67 index of the tumor (4%); however, the pathological specimen of the first tumor was not available. Moreover, it implies regular follow-up, especially between 1 and 5 years after surgery according to the high recurrence rate in this period of time [3].

To the best of our knowledge, it is the first case reported in the literature of a patient who has been affected by recurrent non-functional PA and MTC, concomitantly. Although this association can be accidental, it emphasizes on the fact that patients with a history of a neoplasm should be monitored regularly in order to diagnose and treat possible second primary cancers in a timely manner. Noticeably, this consideration is of great importance in patients whose first neoplasms have better prognosis and survival rates, which provide them more time to develop second primary cancers, e.g., PA.

## Abbreviations

IHC: Immunohistochemical
MEN: Multiple endocrine neoplasia
MTC: Medullary thyroid carcinoma
PA: Pituitary adenoma
TSS: Transsphenoidal surgery
